# Improved Deep Neural Network for Cross-Media Visual Communication

**DOI:** 10.1155/2022/1556352

**Published:** 2022-04-30

**Authors:** Yubo Miao

**Affiliations:** College of Furniture and Art Design, Central South University of Forestry and Technology, Changsha, Hunan 410000, China

## Abstract

Cross-media visual communication is an extremely complex task. In order to solve the problem of segmentation of visual foreground and background, improve the accuracy of visual communication scene reconstruction, and complete the task of visual real-time communication. We propose an improved generative adversarial network. We take the generative adversarial network as the basis and add a combined codec package to the generator, while configuring the generator and discriminator as a cascade structure, preserving the feature upsampling and downsampling convolutional layers of visual scenes with different layers through correspondence. To classify features with different visual scene layers, we add a new auxiliary classifier based on convolutional neural networks. With the help of the auxiliary classifier, similar visual scenes with different feature layers have a more accurate recognition rate. In the experimental part, to better distinguish foreground and background in visual communication, we perform performance tests on foreground and background using separate datasets. The experimental results show that our method has good accuracy in both foreground and background in cross-media communication for real-time visual communication. In addition, we validate the efficiency of our method on Cityscapes, NoW, and Replica datasets, respectively, and experimentally demonstrate that our method performs better than traditional machine learning methods and outperforms deep learning methods of the same type.

## 1. Introduction

Influenced by COVID-19, the home office has become a new office situation, and cross-media visual communication technology has become a key technology for telecommuting. Cross-media visual communication technology is also more common in our life. Thanks to the maturity of 5G technology, current visual communication technologies such as teleconferencing, webcasting, and remote manipulation play an important role in long-distance communication. Nevertheless, current software technologies still have some shortcomings that lead to the loss of the physical connection between the camera and the display, hindering the widespread use of cross-media visual communication systems [1]. Researchers in the literature [2] have tried to solve this problem by upgrading the hardware, but the hardware is too bulky to be mass produced. Some researchers made minor software adjustments and added human eye image correction algorithms to optimize visual communication [3–5].

**Figure 1:**
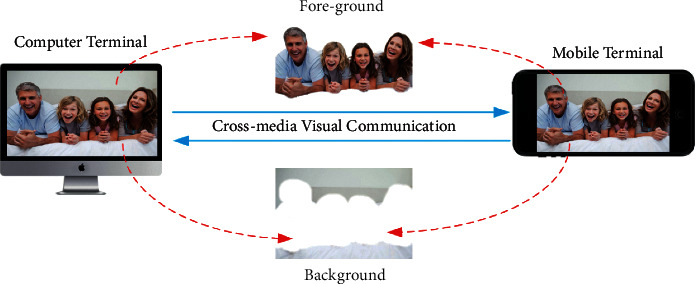
Cross-media visual communication background segmentation.

A cross-media visual communication system often consists of a foreground and a background, with the foreground representing the communicator involved in the visual communication and the background representing scenes other than the communicator [6, 7]. A complete visual communication system is shown in Figure 1. The preliminary visual communication research mainly relies on the collaborative work with stereo cameras to complete the restoration between virtual images to real scenes, and this method requires a high camera scene coverage; in addition, the synchronization and real time between cameras are also required. In later visual communication research, researchers gradually started to use dynamic planning algorithms to achieve control over the matching between virtual scenes by parallax standard lines, as this method could not control the differences between adjacent parallaxes [8–10]. Later, researchers began to explore the rules from the hierarchical algorithm and take advantage of the hierarchical algorithm to exclude the smoothing constraint problem at the parallax end by hierarchical virtual matching layer by layer [11]. Adaptive optimization is also performed for poorer results at the boundaries, and each layer is considered as a uniform set, with each layer corresponding to a pixel depth offset, for parallax compensation. In the image recovery stage, the authors use motion estimation techniques to capture the motion of the virtual image and then match it to the displayed scene [12].

We experimentally verify the efficiency of traditional machine learning methods and deep learning methods in visual communication, and the deep learning methods perform better. So, in this study, we propose a visual communication method based on an improved generative adversarial network. The visual scene is layered by layer features, and foreground and background scenes are trained with unused methods and datasets, respectively. Then, we trained and verified the effectiveness of our method on a public dataset. For the scene segmentation problem of foreground and background, we added control experiments to verify the visual communication efficiency of foreground and background separately with specific datasets. The experimental trials demonstrate that our method is accurate and can achieve real-time performance in the visual communication of foreground and background.

The rest of this study is organized as follows.  Section 2 introduces the research history and research results of cross-media visual communication.  Section 3 describes in detail the principles and implementation processes related to the improved deep visual communication network.  Section 4 shows the related experimental setup, the experimental dataset, and the analysis of the experimental results. Finally, Section 5 summarizes our research and reveals some further research work.

## 2. Related Work

Researchers in the literature [13] have proposed a simulated human eye image correction model to simulate the human eye's ability to process images. The model captures images through a single camera with its position and orientation vectors and then maps the corrected images to the desired cross-media occasions for scene synthesis. In the literature [14], a pairwise polar-constrained view synthesis model is proposed to obtain a wider coverage of visual communication. The authors found in their experiments that the scene coverage of stereo cameras is wider than that of ordinary cameras, so the model uses two independent stereo cameras to capture the scene, and then, the captured model is transformed by 3D point features and mapped to three independent storage units, which can be called directly in scene reduction. Researchers in the literature [15] were inspired by experiments in scene recovery to propose a face model stereo matching algorithm, which has certain requirements for camera placement and requires a pair of simultaneous image captures at the same frame rate phase rate. The virtual camera is then used to synthesize the view, and the synthesized view is matched to the original view for stereo scene matching. Researchers in the literature [16] used a trinocular stereo camera for the first time. The images are captured through the synergy of cameras with different angles, and then, the scene is synthesized by the virtual view, and finally, the depth map is used to recover the communication signal that can support the 3D vision.

In addition to the optimization of the stereo camera matching algorithm, some researchers have discovered a new stereo matching technique, namely, the dynamic planning algorithm [17–19]. This algorithm determines the parallax by scan lines so that the virtual scene matching work can be performed in parallax standard lines. However, this algorithm cannot take into account the differences between adjacent scan lines well, which leads to the error between domains being expanded indefinitely. To address this problem, different dynamic planning algorithms are proposed in the literature [20] according to four different states, respectively. For example, in the case of occlusion, a dual-attention mechanism is invoked to complement the network structure. Besides, the authors also utilize a spatial filtering algorithm to smooth the model, which further improves the stability of the model. The literature [21] is optimized based on the former by adding two new states for segmenting foreground pixels and background pixels to obtain image segmentation capabilities with very high quality. The above research methods are optimized from different perspectives, and all of them eventually improve the matching quality of virtual scenes.

For several years, Markov random fields have been gradually explored by researchers, and their excellent pixel-labeling ability has become a key player in dense stereo matching problems. Some researchers have found in their experiments that the Markov random field is gradually upgraded to a dedicated technique for graph cutting based on the Markov random field in the face of the transformation energy minimization problem, and the main principle of this technique is the confidence propagation minimization law [22–24]. We found in our experiments that the visual communication within and between scan lines is obtained from the Markov random field modeling so that excellent image segmentation capabilities can be obtained in the process of virtual image matching [25–28].

Researchers in the literature [29] found in their model optimization work that the continuity of the model's scene smoothing variability and energy did not satisfy a normal distribution and hypothesized that the reason for the discrepancy was envisioned to be due to the difference in smoothing constraints at the parallax end. In the literature [30], to verify the former idea, a hierarchical stereo matching method was proposed to exclude the smoothing constraint problem at the parallax end layer by layer through hierarchical virtual matching. Also, adaptive optimization is performed for poorer results at the boundaries, and each layer is considered as a uniform set with each layer corresponding to a pixel depth offset for parallax compensation. In the image recovery stage, the authors employ motion estimation techniques to capture the motion of the virtual image and then match it with the displayed scene. The literature [31] innovatively proposed a Bayesian layering method, which concentrates the parallax between layers in a Bayesian decision framework, automatically traverses the parallax without layers by generalized expectation, finds the intermediate value, and finally compensates the parallax beyond the intermediate value. The literature [32] goes deeper in the study of layering, to preserve the boundaries between visual scene boundaries and to ensure the continuity of the boundaries. The authors proposed an iterative layering algorithm to achieve the optimal parallax range by successive iterations of the deep network, and this method somehow improves the real-time processing efficiency.

## 3. Method

### 3.1. Basic Structure

**Figure 2:**
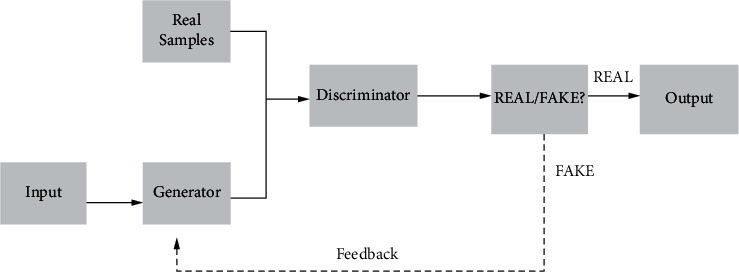
Generative adversarial network architecture.

Generative adversarial networks have great applications in computer vision. Generative adversarial networks are essentially deep neural networks for generative models, which consist of two parts: a generator and a discriminator. The purpose of the network is to simulate learning training samples and generate samples with similar features to the training samples. During the training process, the generator generates a random fake sample from the input sample, and then, the discriminator directly determines the gap between the fake sample and the real sample, and if the gap is beyond the specified range, the fake sample is returned, and the generator regenerates a new sample based on the returned information until the discriminator passes the sample. The structure of the generative adversarial network is shown in Figure 2.

We apply generative adversarial networks to cross-media visual communication. The accuracy of cross-media visual communication is improved by utilizing the mutual game learning approach of this network. Considering the scene complexity and image processing details of our visual communication, adaptive optimization based on generative adversarial networks will be considered to simulate optimal parallax for different visual communication levels. The details of the optimization will be explained in the subsequent subsections.

### 3.2. Generator

**Figure 3:**
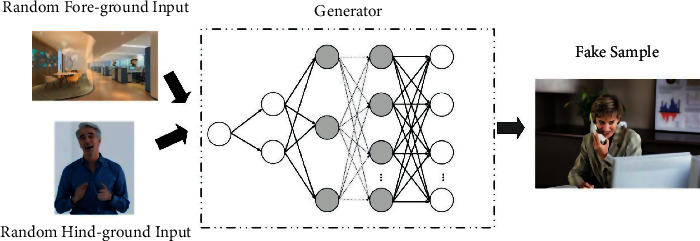
Cross-media visual communication generator process.

For generators, which are usually simple deconvolutional networks or fully connected neural networks, their main purpose is to generate dummy samples based on the output information, and their principle of action is shown in Figure 3. Finally, we usually analyze the generated output with features of different dimensions, and the feature dimensions are not strictly specified before training.

**Figure 4:**
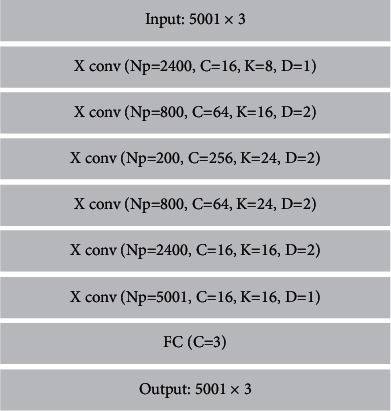
Detailed hierarchy of generators.

The literature [33, 34] propose an improved approach for generating adversarial networks, where the design of the generator as a matching encoder and decoder is very novel and effective. Therefore, we also adopt this combination of coding and decoding packages. We reconfigure the encoding and decoding parts in a cascade structure. The input data encoder, which consists of two convolutional layers, serves for downsampling, and then, *I*^low^ transforms different visual scene data features to the hidden layer. Inspired by the literature [35], we also used a structure of nine residual blocks to enrich different expression intensity features. The decoder consists of two deconvolution layers whose role is to be used for upsampling and to achieve the conversion of intensity features with the target expressions. All the above structures use a step size *s* = 2, kernel = 3 × 3 setting, and all the convolutional layers are followed by a normalization operation and then activated nonlinearly by ReLU. For input surfaces with different expression midpoints, we use the Χ conv operator [36]. For a given *K* input points (*p*_1_, *p*_2_, ..., *p*_*k*_), the *K* input points are weighted by a multilayer perceptron, and then, a *K* × *K*Χ transformation matrix, Χ=*MLP*(*p*_1_, *p*_2_, ..., *p*_*K*_), is performed, followed by an element product summation, and is given to the Χ transform features in as a typical convolution operator treatment. Considering the influence of relative positions between different feature points, we define the Χ conv operator as follows:

(1) $$\begin{array}{c} F_{p}=Χ\_conv\left(K,p,P,F\right), \end{array}$$

(2) $$\begin{array}{c} Χ\_Conv\left(K,p,P,F\right)=Conv\left(K,MLP\left(P-p\right)\times \left[MLP_{\delta }\left(P-p\right),F\right]\right), \end{array}$$

where *p* represents the feature points, *K* represents the adaptive convolution kernel, *P*=(*p*_1_, *p*_2_, ..., *p*_*k*_)^*T*^ represents the *K* points in its neighborhood, and *F*=(*f*_1_, *f*_2_, ..., *f*_*K*_) represents the features of different points. Using the principle of the Χ conv operator, we construct generators representing the parallax lines of visual scenes with different hierarchical structures, as shown in Figure 4. We apply the jump connection in the residual structure to the encoder and decoder to achieve the correspondence of random point coordinate information between the encoder and decoder.

### 3.3. Discriminator

**Figure 5:**
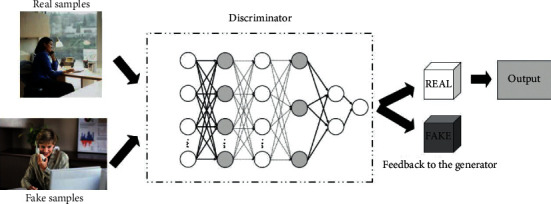
Cross-media visual communication discriminator process.

In the structural design of the discriminator, we also use a combination of a deconvolutional and fully connected neural network designed to distinguish the difference between the fake and real samples generated by the generator. If the difference exceeds a predetermined value, , and if it is trained by fine-tuning the discriminator's samples, it is fed back to the generator by backpropagation. The principle of its action is shown in Figure 5.

**Figure 6:**
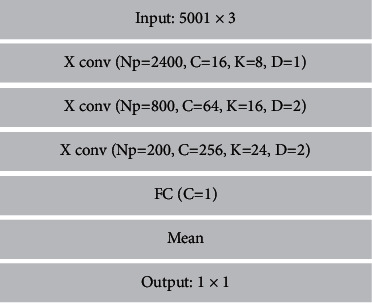
Detailed hierarchy of discriminators.

Low-level visual scene parallax is converted into high-level parallax. We use a generator to filter the underlying data density of high-level visual scene parallax features and a discriminator to separate the visual scene parallax features generated by the generator from the true high-level visual scene parallax features. Inspired by the study in the literature [37], we use alternating training patterns between the generator and discriminator to optimize the discretization problem between maxima and minima. For this purpose, we define the min-max problem as follows:

(3) $$\begin{array}{c} \underset{Gen}{\min}\underset{Dis}{\max}={Ε}_{I}^{high}\log\left(Dis\left(I^{high}\right)\right)+{Ε}_{I}^{low}\log\left(1-Dis\left(Gen\left(I^{low}\right)\right)\right), \end{array}$$

where *Gen* represents the visual scene features generated by the generator, and *Dis* represents the visual scene features determined by the discriminator. {*I*^*low*^, *I*^*high*^} represents a pair of parallax lines with different feature levels but the same visual scene class. The adversarial loss function equations for generator *Gen* and discriminator *Dis* are shown below.

(4) $$\begin{array}{c} L_{G\_a\;dv}=-\frac{1}{N}\sum_{n=1}^{N}\log\left(Dis\left(Gen\left(I_{n}^{low}\right)\right)\right), \end{array}$$

(5) $$\begin{array}{c} L_{D\_a\;dv}=-\frac{1}{N}\sum_{n=1}^{N}\left\{\log\left(Dis\left(I_{n}^{high}\right)\right)+\log\left(1-Dis\left(Gen\left(I_{n}^{low}\right)\right)\right)\right\}, \end{array}$$

where *N* denotes the total number of training samples. In the convergence process, the discriminator converges the features of the fake samples generated by the generator to the features of the real samples by setting the values so that the generator generates rich virtual visual scenes. Similarly, we constructed visual scene discriminators with different hierarchical structures. The hierarchical structure is shown in Figure 6.

### 3.4. Auxiliary Optimization

The scene recognition of different layers of features in similar representations is considered. We introduce auxiliary classifiers in generative adversarial networks for retaining feature information under different layers. In real life, each person is capable of generating hundreds of foregrounds and backgrounds with different meanings and hierarchies in different visual scenes. In our study, the generator is made to enable feature reconstruction of real visual scenes while keeping the visual scene category variables stable. We use the adversarial loss to rationally bootstrap the generator and train the auxiliary classifier to extract visual scene features. Perceptual loss is imposed when optimizing the generator and discriminator.

The auxiliary classifier can efficiently handle highly variable data. To match the adaptive linear fitting neural network, we introduce a convolutional neural network model. The dataset used for classifier training is processed real samples, all of which contain different layers of features. In our experiments, we build an online training method to guarantee its perfect convergence. In the subsequent network phase, we use a feature extraction layer instead of a fully connected layer, and the equation of the perceptual loss function in the feature extraction layer is shown below.

(6) $$\begin{array}{c} L_{perceptual}=\frac{1}{N}\sum_{n=1}^{N}\left\|\phi \left(G\left(I_{n}^{low}\right)\right)-\phi \left(I_{n}^{high}\right)\right\|, \end{array}$$

where *ϕ* denotes the feature extractor, and the biggest role of the perceptual loss function is to capture the variance distance between high-level sensitive features between similar visual scenes, thus maintaining the hierarchical stability of parallax features of visual scenes. In addition, it can also compensate the parallax lines of different layers of features to achieve a steady state.

To contribute to the consistency of the image representation, we use point-by-point loss optimization between the high-level scene representation feature *I*^*high*^ in the real sample and the synthetic virtual scene representation feature *Gen*(*I*^*low*^) in the fake sample. As also mentioned in the literature [38], the distance between the intensity features is constrained by the L1 loss function, while L2 is more efficient in terms of integration performance. Their combined constraint equations are shown below.

(7) $$\begin{array}{c} L_{pixel}=\frac{1}{N_{pixel}}\sum_{i=1}^{N_{pixel}}\left\|Gen{\left(I^{low}\right)}_{i}-I_{i}^{high}\right\|, \end{array}$$

where *N*_*pixel*_ represents the geometric pixels in the two-dimensional sample and also denotes the three-dimensional sample point cloud data points. Combining the above loss functions, the systematic loss function of our optimized generative adversarial network is formulated as follows:

(8) $$\begin{array}{c} L=\omega_{1}L_{G\_a\;dv}+\omega_{2}L_{pixel}+\omega_{3}L_{perceptual}, \end{array}$$

where *ω*_1_, *ω*_2_, and *ω*_3_ represent the weighting coefficients. During the training process, we adopt an alternating training approach so that the generator repeatedly iterates to the optimum and finally generates the sample with the most similar physical signs to the real sample.

### 3.5. Improved Visual Communication Structure

**Figure 7:**
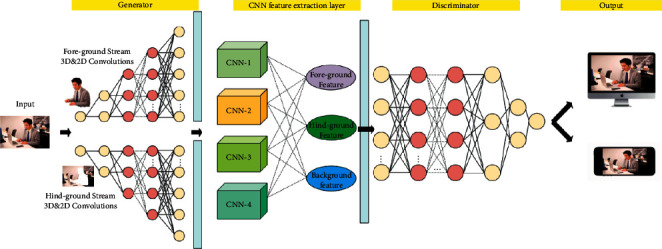
The structure of improved visual communication structure.

To enhance the stability and accuracy of cross-media visual communication systems. We propose an improved deep neural network algorithm for visual communication, and the network underlying the method is a generative adversarial network. With our method, the stability of the cross-mediated visual communication system and the matching accuracy between communication messages can be further enhanced, and a visual scene feature grading model is also used to obtain the optimal parallax lines and compensation values. In our model, the generator synthesizes expressive features with high expression levels under the dual guidance of discriminators and auxiliary classifiers. For 2D samples, the convolutional neural network will assist in generating the samples, and for 3D data, they will be synthesized by the *X* conv operator. The features of different forms of virtual visual scenes are extracted and filtered in the joint output, and the real sample features of the auxiliary classifier are used as the guidance to fuse into a more comprehensive visual scene, so as to achieve an overfitting fit state with the real scene and achieve the maximum matching rate with the real scene. The detailed network structure is shown in Figure 7.

## 4. Experiment

### 4.1. Datasets

To validate the performance of our method, we selected Cityscapes, a public dataset for automatic segmentation of street scenes, NoW, a dataset for 3D face reconstruction, and Replica, a dataset for high-quality 3D reconstruction of indoor scenes, for experimental validation. Before the preprocessing operation on the above datasets, we normalize the visual scene boundaries for all datasets. In the preprocessing stage, the image and video frames are segmented into specified sizes, and different sizes of test methods correspond to different data. We randomly selected 80% of the samples as the training set and 20% as the test set. Next, we describe the three datasets in detail.

The dataset Cityscapes is a large-scale 3D video sequence of urban street scenes, divided into two types of strongly and weakly annotated frames. The biggest advantage of this dataset is its ability to automatically understand the semantics of the scenes and implement pixel-level semantic annotation of the full visual scenes The main advantage of this dataset is that it can automatically understand the scene semantics and achieve full visual scene pixel-level semantic annotation. We mainly choose this dataset to train the model for scene semantic segmentation performance. The dataset NoW is a face reconstruction dataset where all samples are independently performed by a 3D scanner. We mainly choose this dataset to obtain the matching accuracy of communicators in foreground reconstruction and to ensure a clear boundary between foreground and background. The dataset Replica is a high-quality indoor scene dataset, which contains 18 high realistic indoor visual scenes, and its biggest advantage is that all visual scenes are combined at the same level using plane mirrors and reflectors. We mainly choose this dataset to obtain the background matching accuracy and to achieve a high visual background reconstruction rate. The detailed information of the above dataset is shown in Table 1.

### 4.2. Experimental Results

To demonstrate the high efficiency of our method, we compared three methods, random forest (RF), RCNN, and BiLSTM. To ensure the accuracy of each method, each network is independently kept trained without the detection module during the training and tuning process. They are all trained using the ground truth of the three datasets mentioned in the previous section. The deep networks do not use any visual scenes, and among the evaluation metrics, we mainly look at precision (*F*), *F*1 score, and recall (*R*). The detection results of each metric are fed back to the individual metrics in the dataset, and the performance of text detection recognition can be indicated by the connection between the metrics, and the parallax baseline outputs after passing the visual scene classifier, which can also be labeled with the data to react to the accuracy of the virtual scene. There are two main steps in our cross-media visual communication work; first, we perform the visual scene classification work, and then, we perform the virtual scene reconstruction work. The visual scene classification performance is shown in Table 2.

Table 2 shows the visual communication efficiency of our method. The experimental results show that traditional machine learning algorithms poorly perform in visual communication. For example, the random forest algorithm is chosen to represent the traditional machine learning methods in this study, and its visual communication accuracy only reaches 62% at the highest. Among the methods in the deep learning category, RCNN is a better algorithm for visual processing, but its visual scene reconstruction accuracy is not as good as that of the BiLSTM algorithm. This is mainly due to the advantages brought by the special network structure of BiLSTM, which starts from the localization of the real visual scene and the virtual visual scene, and the parallax line compensation from the localization to the whole can optimize the error between adjacent domains well. Second, there is a small-to-large pattern that can expand the visual range and maximize the memory information fusion, which plays a key role in the process of matching the virtual visual scene with the real visual scene. Our method is based on generative adversarial networks, and then, a two-channel model is used to further obtain the bidirectional memory information of the adversarial depth network, which makes the visual communication more accurate.

After the visual scene step, it is difficult to perform the second stage of virtual scene reconstruction work because the traditional machine learning method random forest is not accurate enough in cross-media visual communication. Therefore, in the subsequent experiments, we excluded the traditional machine learning methods and kept only the deep learning methods as the efficiency comparison for visual communication. Before the virtual scene reconstruction work started, we compared three datasets to filter out the most suitable public dataset for cross-mediated visual communication because the data volume was too large. We evaluated the datasets in terms of parallax error rate (PER), virtual scene reconstruction rate (VSRR), and real scene distribution rate (RSDR), and the results of the dataset evaluation are shown in Table 3.

Table 3 shows the performance results of the street scene dataset Cityscapes, the face 3D reconstruction dataset NoW, and the indoor scene reconstruction dataset Replica in our own set of experimental metrics. From the experimental results, we can see that the parallax error rate of street scenes is only 61%, which is a huge difference compared with other datasets. For this problem, we also did the subsequent experimental analysis, and because the complexity of the city street scene is much higher than other datasets, there will be frame loss and feature omission in the scene semantic reconstruction, which indirectly leads to the large error in the parallax of this dataset. At the same time, the Cityscapes data still poorly perform in the virtual scene reconstruction, which is also affected by the complexity of the city street scenes. Given that our cross-media visual communication research is more oriented toward online communication, the scene complexity of the Cityscapes dataset is given. In the subsequent experiments, we will select the NoW dataset and the Replica dataset for experimental validation. Among them, the NoW dataset targets foreground communicators for the scenario experiments. The Replica dataset targets the posterior scenes to verify the matching rate between the reconstructed virtual scenes and the real scenes.

To separate the foreground and background of the cross-media visual communication and highlight the detailed features of the characters, we re-edited the NoW dataset by reconstructing the 3D face reconstruction data in the dataset according to a standardized template and then took a smoothing process for each sample frame. In the scene reconstruction of foreground communicators, we mainly examine the metrics of face matching rate (FMR), foreground segmentation rate (FSR), and pixel offset rate (POR). The experimental results are shown in Table 4.

We also re-edited the Replica dataset and divided the indoor 3D scenes in the dataset into three levels, A, B, and C, where A denotes complexity less than 30%, B denotes complexity between 30% and 70%, and C denotes complexity greater than 70%. Then, a smoothing process is taken for each sample frame. In the scene reconstruction of the background, we mainly examine the metrics of background matching rate (BMR), background segmentation rate (HSR), and pixel offset rate (POR). The experimental results are shown in Table 5.

From the experimental results in Tables 4 and 5, it can be obtained that our method, in the foreground communicator scene reconstruction, has a cross-media visual communication accuracy of 91% and a pixel offset rate of only 5%. In the virtual visual scene reconstruction and real scene matching in the back view, the cross-media visual communication rate reaches over 80% in the most complex indoor scene, and the pixel offset rate is up to 9%, which satisfies the error controllable range. Meanwhile, the data transmission rate is between 55 fps and 56 fps, and the cross-media visual transmission of both foreground and background meets the real-time requirements of visual communication.

## 5. Conclusions

To improve the accuracy and real-time performance of cross-media communication, we propose an improved generative adversarial network. Considering the cascade structure of the deep adversarial network will be useful for cross-mediated visual communication. Therefore, we choose the generative adversarial network as the basis and add a combined codec packet to the generator, while configuring the generator and discriminator as a cascade structure, preserving the feature upsampling and downsampling convolutional layers of visual scenes with different layers through correspondence. To classify features with different layers of visual scenes, we added a new auxiliary classifier based on convolutional neural networks. With the help of the auxiliary classifier, similar visual scenes with different feature layers have more accurate recognition rates. In the experimental part, we validate the efficiency of our method on Cityscapes, NoW, and Replica datasets, respectively, and experimentally demonstrate that our method performs better than traditional machine learning methods and outperforms deep learning methods of the same type. To better distinguish between foreground and background in visual communication, we perform performance tests on foreground and background with separate datasets. The experimental results demonstrate that our method has excellent accuracy in both the foreground and background of cross-media communication and can achieve real-time visual communication.

Cross-media visual communication is an extremely complex task that encompasses scene reconstruction, scene matching, and foreground-background segmentation. Each application scene has thousands of foreground-background combinations. In this study, we choose the simpler indoor scenes as our research point. However, for real cross-media visual communication scenes will be more complex. For complex visual scenes, our approach still poorly performs. In the next study, we will try to use the local perceptual layer of the BiLSTM network as the implicit layer visual scene extraction point to increase the visual scene capture range and improve the coverage of the model for visual scene types.

## Figures and Tables

**Table 1:** The detailed information of datasets.

	Datasets		
	Cityscapes	NoW	Replica
Train	2301	3025	2361
Test	756	498	530
Total	3057	3523	2891

**Table 2:** Results of visual communication by different methods.

	Cityscapes			NoW			Replica		
	*P*	*R*	*F*1	*P*	*R*	*F*1	*P*	*R*	*F*1
RF	0.58	0.56	0.61	0.62	0.66	0.62	0.62	0.55	0.63
RCNN	0.70	0.76	0.70	0.73	0.59	0.66	0.73	0.63	0.65
BiLSTM	0.78	0.83	0.83	0.77	0.73	0.74	0.85	0.79	079
Ours	0.91	0.93	0.86	0.89	0.85	0.86	0.90	0.92	0.90

**Table 3:** Differences in visual communication between different datasets.

	Cityscapes	NoW	Replica
PER	0.61	0.87	0.92
VSRR	0.69	0.80	0.86
RSDR	0.76	0.88	0.84

**Table 4:** Experiment results of foreground dataset.

	NoW		
	Train	Test	Fps
FMR	0.90	0.91	55
FSR	0.88	0.91	
POR	0.05	0.03	

**Table 5:** Experiment results of background dataset.

	Replica			
	A (0–30%)	B (30%–70%)	C (70%–100%)	Fps
BMR	0.96	0.90	0.83	56
HSR	0.97	0.88	0.80	
POR	0.02	0.05	0.09	

## Data Availability

The dataset can be accessed upon request.
